# Evaluation of STIR Library Adapted for PET Scanners with Non-Cylindrical Geometry

**DOI:** 10.3390/jimaging8060172

**Published:** 2022-06-18

**Authors:** Viet Dao, Ekaterina Mikhaylova, Max L. Ahnen, Jannis Fischer, Kris Thielemans, Charalampos Tsoumpas

**Affiliations:** 1 Leeds Institute of Cardiovascular and Metabolic Medicine, University of Leeds, Leeds LS2 9JT, UK; c.tsoumpas@umcg.nl; 2 Positrigo AG, 8005 Zurich, Switzerland; ekaterina.mikhaylova@positrigo.com (E.M.); max.ahnen@positrigo.com (M.L.A.); jannis.fischer@positrigo.com (J.F.); 3 Institute of Particle Physics, ETH Zurich, Otto-Stern-Weg 5, 8093 Zurich, Switzerland; 4 Institute of Nuclear Medicine, University College London, London NW1 2BU, UK; k.thielemans@ucl.ac.uk; 5 Centre for Medical Image Computing, UCL, Gower Street, London WC1E 6BT, UK; 6 Algorithms Software Consulting Ltd., London SW15 5HX, UK; 7 Department of Nuclear Medicine and Molecular Imaging, University Medical Center Groningen, University of Groningen, 9713 GZ Groningen, The Netherlands

**Keywords:** tomography, image reconstruction, brain, imaging

## Abstract

Software for Tomographic Image Reconstruction (STIR) is an open source C++ library used to reconstruct single photon emission tomography and positron emission tomography (PET) data. STIR has an experimental scanner geometry modelling feature to accurately model detector placement. In this study, we test and improve this new feature using several types of data: Monte Carlo simulations and measured phantom data acquired from a dedicated brain PET prototype scanner. The results show that the new geometry class applied to non-cylindrical PET scanners improved spatial resolution, uniformity, and image contrast. These are directly observed in the reconstructions of small features in the test quality phantom. Overall, we conclude that the revised “BlocksOnCylindrical” class will be a valuable addition to the next STIR software release with adjustments of existing features (Single Scatter Simulation, forward projection, attenuation corrections) to “BlocksOnCylindrical”.

## 1. Introduction

**Figure 1:**
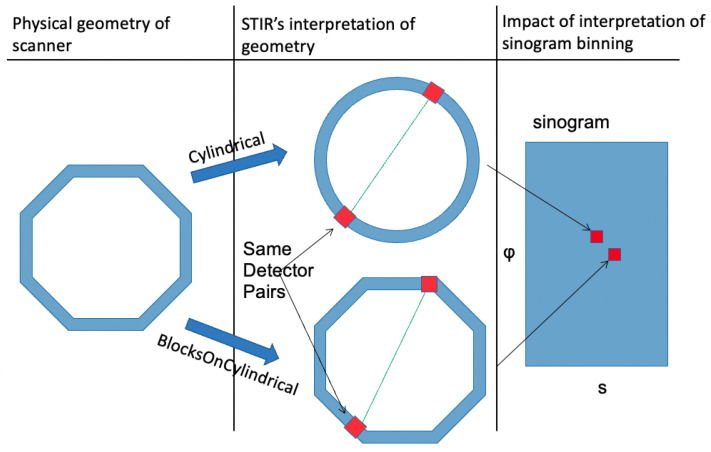
An illustration of STIR’s new class using one LOR. First column is the physical geometry of the scanner. Second column is STIR’s interpretation of such geometry using different geometry class: top is “Cylindrical” class, bottom is “BlocksOnCylindrical” class. Each class interprets each LOR from the same detector pairs differently due to different positions of the detector pairs. The difference in the position of the detector pairs causes the calculation of the LOR to be different in both (s,ϕ) coordinates.

A dedicated organ positron emission tomography (PET) scanner has the ability for higher spatial resolution and increased sensitivity [1] compared to conventional PET because it is closer to the source. One such example of a dedicated organ PET scanner is the high-resolution research tomograph (HRRT) dedicated brain scanner built by CTI. The HRRT PET has a spatial resolution of 2.5 mm [1]. However, when closer to the body, geometry modelling requires careful revision and adaptation in software. The system matrix can be separated into different components, such as the geometry of the scanner, image-based blur effect, and positron range specified by Rahmim et al. [2] (p. 5949). This study will focus on the system matrix related to the scanner’s geometry. Previous work has been implemented in geometry modelling for PET, such as PRESTO [3] and CASToR [4]. PRESTO’s implementation is to project the line of response (LOR) from non-cylindrical geometry to cylindrical geometry, whereas CASToR allow users to defined their own classes to suit their needs. In this study, we shall focus on a software package called STIR [5]. The STIR software is a toolkit that allows users to perform image reconstruction, data conversion (e.g., listmode to projection), and data correction (randoms, scatter, normalisation) with PET and SPECT data. STIR has a large community with much support for future updates. Traditionally, STIR approximates the scanner geometry as virtual detectors placed on the cylinder with two open ends. As a result, the scanner’s detectors are equally spaced in the transaxial and axial directions. However, many scanners’ geometry is a polygonal prism, not a cylinder. This inaccuracy in modelling geometry causes misplacement of endpoints in the LOR and, therefore, an increase in uncertainty in the coordinate system of the measurement (noted as (s,ϕ) as shown in Figure 1). This uncertainty can result in significant artefacts in the reconstructed images.

The “BlocksOnCylindrical” class is designed for better modelling of the system matrix. There are alternative methods to estimate the system matrix using empirical methods and/or Monte Carlo simulations, e.g., Geant4 [6]. However, both methods are slow, burdened with statistical noise time, and laborious (moving the mechanical source position), as stated by Iriarte et al. [7] (p. 36). “BlocksOnCylindrical” overcomes this by generating the system matrix on the fly.

The system matrix related to the scanner geometry can be calculated on the fly while performing Siddon ray tracing [8] at the endpoint of each LOR using more accurate modelling of each detector’s position. Therefore, this is an analytical method, which helps reduce the computational time compared to the empirical methods and Monte Carlo simulations.

Figure 1 shows two virtual scanner geometries, which differ based on STIR’s interpretation of geometry class, of a real non-cylindrical scanner and how it impacts the LOR position in the sinogram. The use of the “Cylindrical” class produces a different bin position than the ”BlocksOnCylindrical“ class. The mismatch between real and virtual scanner geometry causes the LOR to have different (s,ϕ) coordinates despite the identical detector pairs. This misalignment in coordinates is the cause of the line artefact [9]. Overall, Khateri et al. [9] concluded that the use of the ”BlocksOnCylindrical” class for scanner geometry studied by the author improves the spatial resolution of the reconstructed image. Specifically, the spill-over ratio for a 1.8 mm diameter rod is improved from 0.34 to 0.19 [9] (pp. 9–10), and the coefficient of variation (standard deviation divided by mean) is approximately 15% lower for block compared with cylinder without normalisation [9] (p. 10 Table 2). These results justify a study of a potential application of the “BlocksOnCylindrical”.

In this study, we test the experimental “BlocksOnCylindrical” class, diagnose problems, and try to improve STIR by using measured and simulated data from a real octagonal prism dedicated PET scanner called Brain PET (BPET) [10].

The aims of this study are:

1. Diagnose the “BlocksOnCylindrical” class using various phantoms (measured or simulated) as test objects for the BPET scanner;
2. Improve the “BlocksOnCylindrical” class for more accurate reconstructions;
3. Incorporate various features (single scatter simulation, attenuation correction, forward projection), previously only available in the “Cylindrical”class, into the “BlocksOnCylindrical” class;
4. Perform quantitative measurements using various phantoms, such as a point source (spatial resolution), a uniform cylinder (uniformity), and an image quality phantom as proposed by Moliner et al. [11].

## 2. Materials and Methods

### 2.1. Materials

#### 2.1.1. PET Scanner

**Figure 2:**
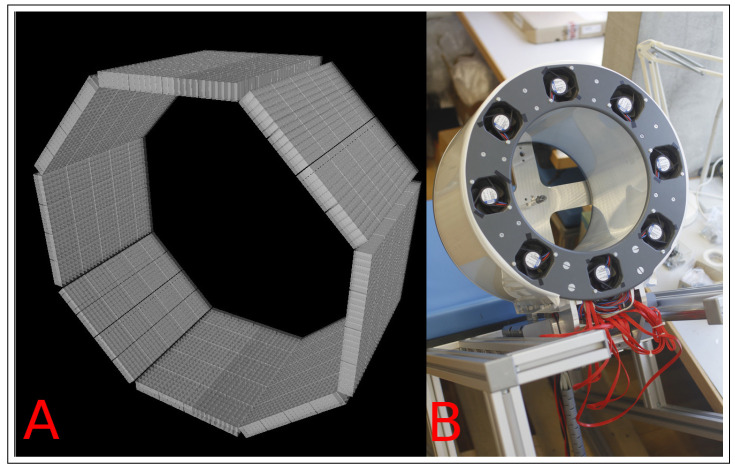
Left (**A**): computer render of the crystal layout. Right (**B**): physical BPET scanner attached to a chair.

In this study, we apply the “BlocksOnCylindrical” class to image reconstructions and corrections of the data obtained by the BPET scanner [10]. BPET detectors are arranged into an octagonal prism forming eight ring sectors. Each sector consists of four detector blocks in the transaxial direction and five in the axial direction. Each block is an array of 6 × 6 LYSO crystals, and each crystal has a size 4.1 mm × 4.1 mm × 10 mm. This configuration is shown in Figure 2.

#### 2.1.2. Phantoms

The first phantom is a Monte Carlo simulation of two point sources placed at the centre of the axial field of view (FOV) in two different positions in the scanner’s transaxial FOV offset: (1) at 10 mm, (2) at 100 mm.

The corresponding sinograms and the reconstructed images are obtained and compared. In addition, the sinograms allow us to compare the difference between the Monte Carlo simulation and STIR’s sinogram output.

The next phantom is a uniform cylindrical phantom. The simulation phantom radius is 108 mm and is used to extract true coincidences only to perform diagnostics on the “BlocksOnCylindrical” class. The measured uniform cylindrical phantom is uniformly filled with [F18] FDG water solution of 5 MBq total activity. The phantom’s radius is 108 mm, and the length extends through the entire axial FOV of the scanner. The phantom was centred axially and transaxially in the FOV and scanned for five hours. The measured uniform cylinder is used for the normalisation procedure.

**Figure 3:**
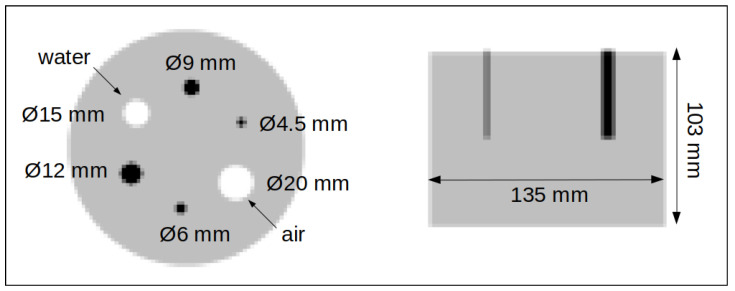
Geometry of the image quality phantom [11]. The phantom and four smaller rods were filled with F18 water. One rod was filled with non-radioactive water, and the biggest rod was left empty. The hot rod to background activity concentration equals 4-to-1.

For the third study, a measured image quality phantom consisting of several radioactively “hot” and “cold” rods placed in a “warm” background is used [11]. The phantom geometry, dimensions, and the positions of the rods are shown in Figure 3, which summarises the filled-in materials, diameters, and the ratios of rods’ activity to the background. In addition, this phantom is used for quantitative measurement (NEMA NU 2-2012 [12]).

#### 2.1.3. Metrics

There are three main metrics used in this report: full width at half maximum (FWHM), uniformity, and contrast. First, the two point sources are used to measure spatial resolution along the three dimensions. The uniform cylinder uniformity is characterised by the coefficient of variation (COV). Finally, the image quality phantom is used to calculate percent contrast for each hot and cold rod in the radioactive water background according to the NEMA NU 2-2012 standard.

### 2.2. Methods

#### 2.2.1. Monte Carlo Simulations

A part of our testing was done using data simulated with the open source GEANT4 Application for Tomographic Emission (GATE) software (version v9.2) [13], specifically, the point sources and the uniform cylinder acquisitions. The GATE configuration used did not model the positron range or the non-colinearity of annihilation photons. The important physical effects such as gamma attenuation and Compton scattering in phantoms and detector crystals were modelled. The simulated coincidence data were saved after applying 425–650 keV energy window.

#### 2.2.2. Iterative Reconstruction Pipeline

The reconstruction we use is the maximum likelihood expectation–maximisation algorithm, also known as MLEM. MLEM was run for 40 iterations for each of the phantoms. During each iteration, correction factors (normalisation, scatter, random, and attenuation) were applied as multiplication and additive factors as described below.

The fraction of gamma photons detected by a PET detector is known as the efficiency of the detector. This is dependent of the detector properties and geometry of the scanner. The variance in detector efficiencies can be modelled by measuring sufficient coincidences for each detector pair. The normalisation phantom used in this work was scanned for 5 h. The measured normalisation data are corrected for scattering (using single scatter simulation in full resolution mode) and randoms. We used a component-based normalisation and expectation–maximisation algorithm, which is available in STIR [14]. This method assumes two components: crystal efficiency factors and geometrical factors, the latter obeying the symmetry of the scanner geometry.

Attenuation of gamma photons is a process of gammas being absorbed by the medium through which they travel. The probability of gammas being attenuated depends on the density of the medium and the travel distance. The attenuation of photons in PET causes loss of counts and severe deterioration of small features in the inner parts of the imaged object. The data sinogram is multiplied by the sinogram of the inverse of the attenuation factors. This process requires the attenuation map to be known. For simple phantoms, the attenuation image can be generated in STIR, and attenuation correction factors were derived from such image.

A random coincidence happens when two gamma photons from two different annihilation events are registered as a coincidence. Similar to scattered events, this causes a false coincidence. In this study, we estimate the contribution of random coincidences from single events recorded during each scan [15].

Compton scattering is a phenomenon where photons interact with atoms and change their original trajectory as a result. To assess the contribution of the scattered coincidences to the measured data, we used STIR’s utility called scatter estimation. The data (corrected for attenuation and randoms) are reconstructed, and then Compton scattering is derived from this image using the single scatter simulation, described by Tsoumpas et al. [16]. The scatter profile was then checked for a tail fit using the method described by Thielemans et al. [17]. This process was performed for several iterations and averaged.

### 2.3. STIR Development

Throughout this study, there were some changes in STIR. One significant development is a better calculation of the forward projector for a more accurate reconstruction and the adjusted single scatter simulation (SSS) for the “BlocksOnCylindrical” class.

#### 2.3.1. Forward Projection

Traditionally, the forward projector of STIR works with the “Cylindrical” geometry class. The calculation for the s-coordinate of the “Cylindrical” class is s=r×sin(θ), where *r* is radius of scanner. This calculation assumes that scanner geometry is cylindrical due to the radius being in the calculation. Therefore, it performs ray tracing at the wrong detector coordinates when calling for the “BlocksOnCylindrical” geometry class. The proposed solution to this is to calculate the *s*-coordinate using vectors. The method gives a general calculation of *s*-coordinate compared to the previous calculation, and it requires:

1. The coordinates of two endpoints of LOR;
2. The origin of the coordinate system to be set to the centre of the scanner, i.e., origin is at $\left(0,0,\frac{l}{2}\right)$, where *l* is scanner length.

**Figure 4:**
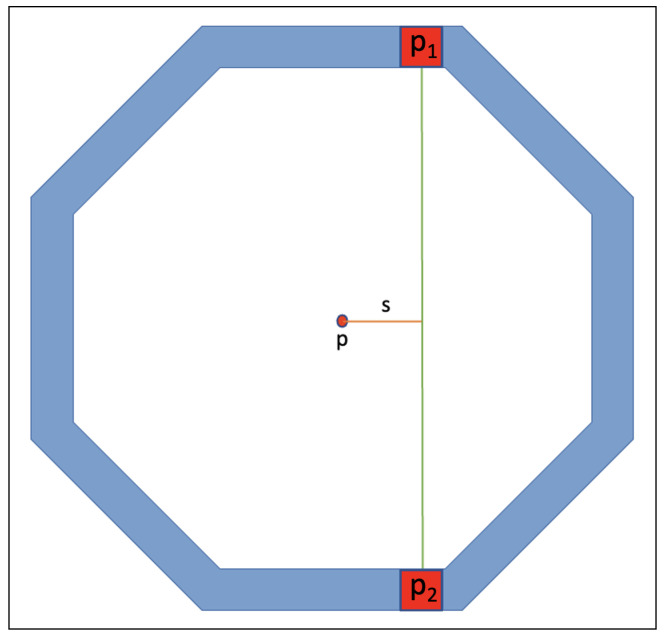
Calculation of s-coordinate using vectors where *p* is the origin, and $p_{1}$ and $p_{2}$ are endpoints of LOR. The s-coordinate corresponds to the normal distance from p to LOR (line from $p_{1},p_{2}$).

The value for the new s-coordinate is

$$s=\left|\right|\left(p-p_{1}\right)-\frac{\left(p-p_{1}\right)\cdot \left(p_{1}-p_{2}\right)}{\left|\right|p_{2}-p_{1}{\left|\right|}^{2}}\left(p_{2}-p_{1}\right)\left|\right|$$

where *p* is the origin, $p_{1},p_{2}$ are the two LOR endpoints and · is a dot product between two vectors. An illustration of this equation is shown in Figure 4.

#### 2.3.2. Single Scatter Simulation

Similar to the forward projector, the single scatter simulation (SSS) is designed for the “Cylindrical” class. Therefore, there was a mismatch between scatter only coincidence extract from GATE and STIR SSS in the case of the “BlocksOnCylindrical” class. Therefore, an improvement was applied to allow changes in the geometry (“Cylindrical” or “BlocksOnCylindrical”) during the initialisation of SSS.

## 3. Results and Discussion

### 3.1. Forward Projection

**Figure 5:**
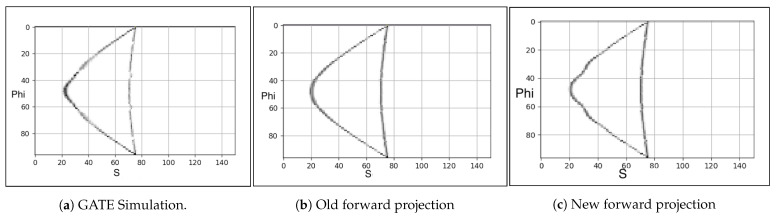
Forward projection of two point sources at the axial FOV centre and at the 10 mm and 100 mm offset from the transaxial FOV centre: GATE (**a**), STIR’s old calculation (**b**), STIR’s new calculation (**c**).

Following the changes to the forward projector in Section 2.3.1, we tested its performance using point sources. The point sources were simulated in GATE, then processed and output as listmode data, using in-house software, which are then converted to projection data (Figure 5a). Figure 5c is a sinogram constructed from STIR’s forward projector using the new *s*-coordinate, and it closely resembles the GATE simulation in Figure 5a when compared to the forward projection of point source using the previous calculation of *s*-coordinate shown in Figure 5b.

**Figure 6:**
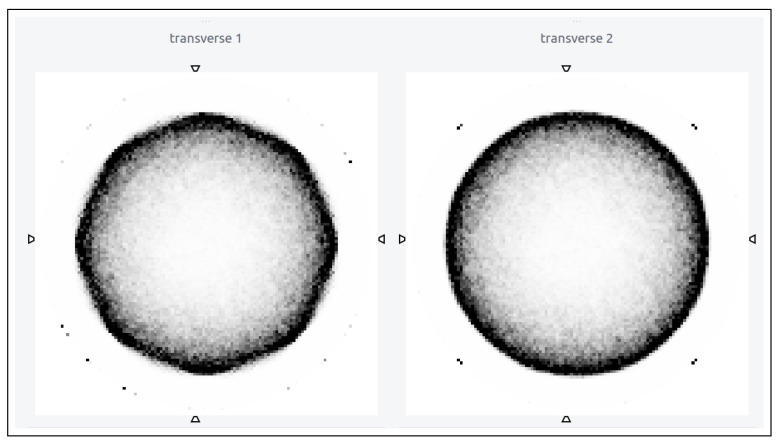
Transverse planes of the reconstructed images of a uniform cylinder simulated in GATE using the “BlocksOnCylindrical” class, with only true coincidence and no data correction with: old forward projector (**left**) and new amended forward projector (**right**).

Other classes that call the forward projector are automatically updated for the “BlocksOnCylindrical” case, e.g., derivation of the attenuation correction factor sinograms from a given attenuation map. The left-hand side of Figure 6 shows the cross-sectional images obtained by reconstruction of the simulated uniform cylinder without any improvement to the s-coordinate of the forward projector, whereas the right-hand side of Figure 6 shows the same obtained with the more accurate calculation of the s-coordinate. The octagonal shaped artefacts (left Figure 6) have virtually disappeared (right Figure 6) with this improvement.

### 3.2. Single Scatter Simulation

**Figure 7:**
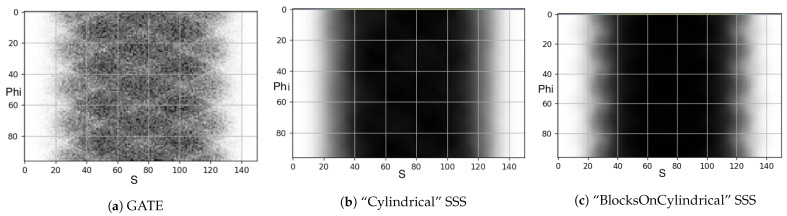
Scatter simulation of the uniform water cylinder: scatter coincidences extracted from the GATE simulations (**a**), STIR’s “Cylindrical” class SSS (**b**), and STIR’s “BlocksOnCylindrical” class SSS (**c**), whose sinogram shape closely matches the shape of the GATE-produced scatter sinogram (**a**) when compared to the sinograms shape computed using the “Cylindrical” class SSS (**b**).

We also tested the performance of the SSS algorithm implemented in the STIR software for the uniform cylinder simulated in GATE. The scattered coincidences are extracted from the simulation and converted into a sinogram (Figure 7a). Similarly, we created an identical uniform cylinder in STIR and performed SSS using the “Cylindrical” and “BlocksOnCylindrical” classes. The results are shown in Figure 7. Figure 7c resembles the GATE simulated results shown in Figure 7b when compared to the “Cylindrical” case SSS (Figure 7b).

### 3.3. Up-Sampling of Single Scatter Simulation

**Figure 8:**
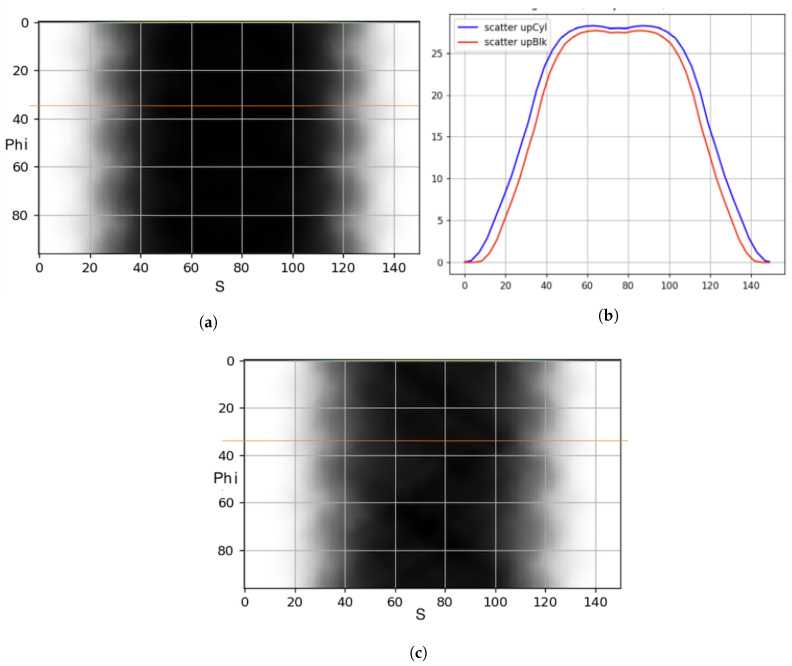
“BlocksOnCylindrical” SSS: using full resolution (top left) and upsampling (bottom left). Line profile of the orange line (shown in sinogram of SSS) of full resolution SSS (blue line) compared to the up-sampled low resolution SSS (red line). (**a**) Full resolution BlocksOnCylindrical SSS. (**b**) Line Profile. (**c**) Upsampled BlocksOnCylindrical SSS.

Single scatter simulation is generally executed with fewer detectors and fewer rings than the actual scanner (in our approach, we downsampled by half rings and half detectors) and after the simulation, the sinograms are up-sampled to the size of the actual scanner by interpolation. Upsampling enables SSS to be computationally efficient. Unfortunately, up-sampled SSS is inaccurate when calculated for the non-cylindrical PET scanner. Wrong upsampling is noticed when full resolution SSS (using the new geometry) is performed and compared with up-sampled low-resolution SSS using line profiles (Figure 8b).

Figure 8 shows the upsampled low-resolution SSS has a much tighter width compared to full resolution SSS. One potential cause of this issue is the single slice rebinning (SSRB) algorithm implemented only for the “Cylindrical” scanner geometry. The temporary solution for this problem is to skip upsampling and perform full resolution 2D (no oblique sinograms) SSS. SSRB will need an appropriate update to work with any geometry.

### 3.4. Spatial Resolution

The FWHMs for the GATE simulated point sources are listed in Table 1.

**Figure 9:**
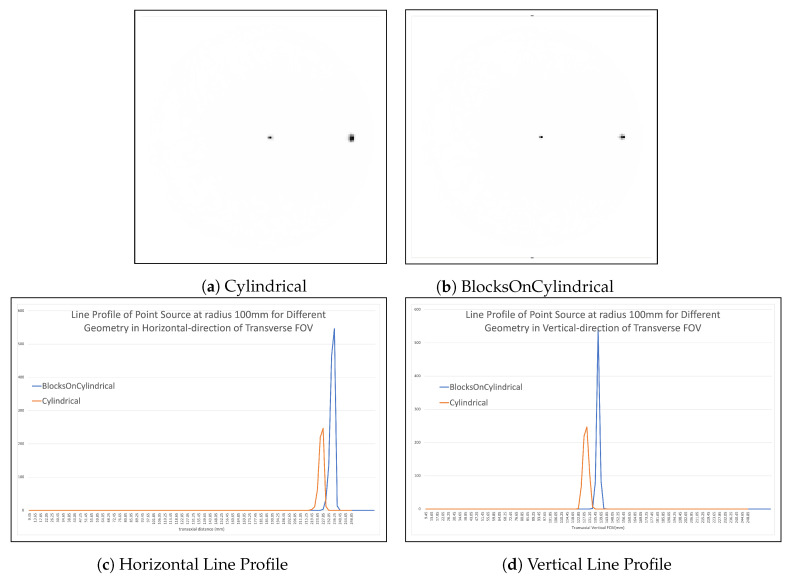
Reconstruction of two sources using “Cylindrical” (**a**) geometry and “BlocksOnCylindrical” (**b**). Line profile for the point source at 100 mm radius in the horizontal direction (**c**) and vertical direction (**d**).

As shown in Table 1, there is an improvement in the point spread function FWHM in all directions and for all point sources when the “BlocksOnCylindrical” class is used for the forward projection step of the image reconstruction algorithm. In particular, there is a significant improvement in FWHM for “BlocksOnCylindrical” in the axial direction compared to “Cylindrical”, from 3.44 mm to 2.96 mm. There is a larger improvement for the point source closer to the edge of the FOV in the y-direction, from 5.1 mm to 2.48 mm. The transverse views of the images produced by the two models of the reconstruction are shown side by side in Figure 9a,b. The corresponding line profiles placed horizontally and vertically of the point source at 100 mm are shown Figure 9c,d.

The reduction in FWHM can be attributed to better modelling of the detector position and, therefore, better estimation of the system matrix related to the scanner geometry. It should be noted that any measurements taken using the point source were from the GATE simulation, where the length of the crystals was set to 1 mm to minimise the effect of the depth of interaction caused on the resolution. Furthermore, the simulation was also performed without any positron range or photon non-colinearity, which is why the FWHM is small.

### 3.5. Uniformity Measurements

The cylinder uniformly filled with radioactive F18 water was scanned for 5 h with BPET. The coefficient of variation (COV) was calculated within a cylindrical region of interest (ROI) with the radius of 10 cm and the length of 5 cm drawn in the centre of the uniform cylinders. The results are shown in Table 2.

**Figure 10:**
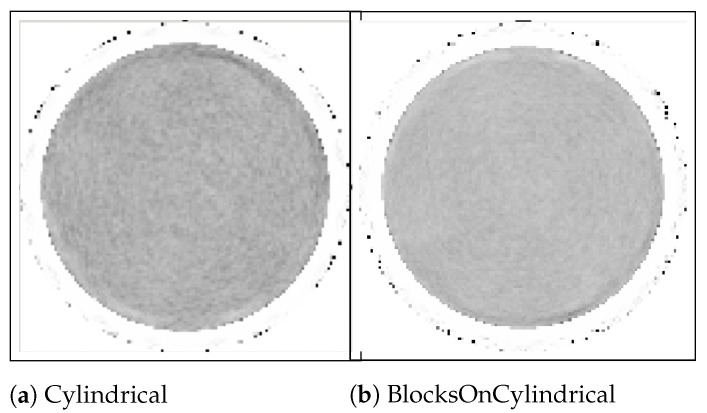
Reconstruction of the uniform cylinder using the “Cylindrical” class (**a**) and the “BlocksOnCylindrical” class (**b**).

Table 2 illustrates that there is a slight increase in mean value, whereas the COV reduces by 10% when the “BlocksOnCylindrical” class is used for the image reconstruction and data correction. The central slice of the reconstructed uniform cylinder compared for the “BlocksOnCylindrical” and “Cylindrical” classes is shown in Figure 10.

This decrease in COV, as shown in Table 2 and Figure 10, is a direct consequence of more accurate modelling of the detectors’ positions by the “BlocksOnCylindrical” class.

### 3.6. Contrast Measurements

**Figure 11:**
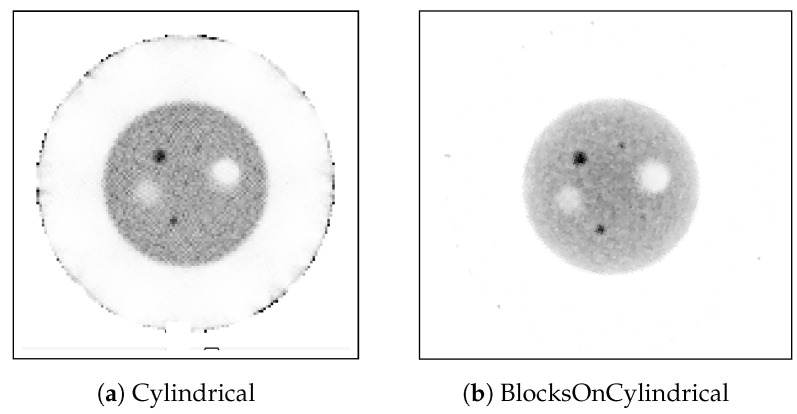
Transverse view of reconstructed image quality phantom produced by the: (**a**) “Cylindrical” class with all corrections, (**b**) “BlocksOnCylindrical” class with all corrections.

The cross-sectional images shown in Figure 11 illustrate the difference between the image reconstruction results produced by the “Cylindrical” and the “BlocksOnCylindrical” classes when applied to the phantom data (Section 2.1.2) measured by the BPET scanner.

The reconstruction using the “Cylindrical” class contains a false small hot rod right in the transaxial centre of the FOV (Figure 11a), as well as does not show the 4.5 mm hot rod. Additionally, the image is grainy (higher COV, discussed in uniformity). In contrast, Figure 11b does not contain any false hot rod in the FOV centre, and the 4.5mm rod is visible and has a more uniform background than in Figure 11a. Finally, we performed a quantitative evaluation of the contrast shown in Table 3, where it can be noticed that all active rods have an increase in contrast anywhere from 2.9% (active rod with 6 mm diameter) to 19.3% (active rod with 12 mm diameter).

### 3.7. Outlook

In this investigation, we demonstrated that we amended the software library STIR to allow the reconstruction of improved images. Additional components we envisage incorporating in the future are a model for the depth of interaction and the detector response function [2], which are not implemented in STIR for user defined geometries in the form of a detector ID map used in binning. Furthermore, we envisage that users can define their own geometry from such crystal maps, and STIR will perform reconstruction and correction using such crystal maps. Development has already started, and it is called the “Generic” geometry class.

## 4. Conclusions

We demonstrated tests and improvements to STIR to work harmoniously with “BlocksOnCylindrical” for a brain PET scanner. We resolved a geometric artefact (octagonal uniform cylinder) using a more generic calculation of the *s*-coordinate in the forward projector. We retrofitted features previously available to the “Cylindrical” class to the “BlocksOnCylindrical” class. We identified a noticeable improvement in spatial resolution, uniformity, and contrast from “Cylindrical” to “BlocksOnCylindrical”, which agree with the Khateri et al. [9] findings. The “BlocksOnCylindrical” feature shows a promising future in STIR. Apart from SSRB adaptations, all these features are planned for STIR 5.0, to be released in 2022.

## Figures and Tables

**Table 1:** FWHM of two simulated point sources; “a”, “t” stands for axial and transaxial; X, Y, and Z follows the LPS coordinate where: X is from right to left, Y is anterior toward posterior, and Z is from inferior to superior.

Source Location	Cylindrical			BlocksOnCylindrical		
in mm	FWHM	FWHM	FWHM	FWHM	FWHM	FWHM
	X	Y	Z	X	Y	Z
	(mm)	(mm)	(mm)	(mm)	(mm)	(mm)
a: 0, t: 10 mm	2.21	2.24	3.43	2.15	2.18	2.96
a: 0, t: 100 mm	2.22	5.10	4.26	2.14	2.48	4.18

**Table 2:** Mean, standard deviation (SD), and coefficient of variation (COV) of the measured uniform cylinder.

Measurement	Cylindrical	BlocksOnCylindrical
Mean	1.75	1.78
SD	0.26	0.23
COV	0.15	0.13

**Table 3:** Image quality phantom: cold ^a^ and hot ^b^ rod contrast [12].

Rod	Cylindrical		BlocksOnCylindrical	
Diameter, mm	Contrast, %	Background Variability, %	Contrast, %	Background Variability, %
20 ^a^	78	4.9	75	4.8
15 ^a^	52	2.2	71	2.2
12 ^b^	35	2.2	54	2.2
9 ^b^	24	2.2	28	2.2
6 ^b^	13	2.2	16	2.2

## Data Availability

Contact authors for dataset.
